# Gradient Structure Construction of High Thermal Conductivity Polyurethane/Boron Nitride Composite Fiber Membrane for Thermal Management

**DOI:** 10.3390/molecules30071449

**Published:** 2025-03-25

**Authors:** Zhengyang Miao, Jingwei Li, Yidan Liu, Fang Jiang

**Affiliations:** 1 State Key Laboratory of Bio-Based Fiber Materials, Zhejiang Sci-Tech University, Hangzhou 310018, China; mzy1147911302@163.com (Z.M.); ljw1263360689@126.com (J.L.); liuyidan@zstu.edu.cn (Y.L.); 2 Shaoxing-Keqiao Institute, Zhejiang Sci-Tech University, Shaoxing 312000, China; 3 Faculty of Science, Shanghai University, 99 Shangda Road, Shanghai 200444, China

**Keywords:** thermal management, thermal conductivity, mechanical property, gradient structure

## Abstract

Accompanied by the rapid progress of the digital era and the continuous innovation of material science and technology, wearable electronic devices are widely used in various industries due to their excellent portability and flexibility. However, the problem of heat accumulation not only restricts the use of electronic devices but also poses potential safety risks for users. Therefore, there is an urgent need to study and develop thermal management materials applied to wearable devices to meet the demands of highly integrated wearable electronic systems. In this study, we report a method of combining functional boron nitride (FBN) and polyurethane (PU) through electrostatic spinning technology and gradient structure design, which ultimately results in multilayer structured FBN/PU composite fiber membranes with excellent thermal conductivity (2.96 W·m^−1^·K^−1^) and mechanical properties (The tensile strength, Young’s modulus, and toughness were up to 12.03 MPa, 86.37 MPa and 15.02 MJ·m^−3^, respectively). The gradient structure design significantly improves the thermal conductivity and mechanical properties of the composite fiber membrane. The multilayer structured composite fiber membrane has high thermal conductivity and high mechanical properties and has potential application and development prospects in the thermal management of wearable electronic devices.

## 1. Introduction

The development of science and technology improves wearable electronic devices, making them more useful and common in various sectors, such as biomedicine, signal detection, and energy storage, due to their excellent portability and flexibility [1,2,3,4,5]. However, the prolonged operation of electronic devices inevitably triggers the generation and accumulation of large amounts of heat inside the devices, and the accumulation of heat may not only cause irreversible damage to the service life of the devices but also pose potential safety risks to the users. Dealing with heat accumulation quickly and efficiently has turned out to be a core aspect of promoting the development of wearable electronic devices [6,7,8,9,10]. Therefore, there is an urgent need to study and develop thermal management materials with a combination of high thermal conductivity, flexibility, and mechanical properties that can be used in wearable devices to meet the demands of highly integrated wearable electronic systems [11,12,13]. Thermal management materials are usually prepared by introducing thermally conductive fillers into the polymer matrix to improve thermal conductivity, but the limitations of the filler sheets lead to a reduction in the mechanical properties of the polymers, which makes it difficult to meet the needs of wearable electronics.

The construction of thermally conductive pathways is a very important issue in improving the thermal conductivity of boron nitride (BN) composites, which can be achieved by the orientation and selective distribution of the fillers in the polymer matrix [14,15,16]. Zhang et al. [17] reported a strategy of chemical–physical double cross-linking and tensile orientation, constructing highly ordered cellulose nanofiber networks and edge-hydroxylated boron nitride nanosheet (BNNS-OH) structures, which induced the in-plane orientation of BNNS-OH to achieve high tensile strength and in-plane thermal conductivity. Song et al. [18] reported an aerogel film with a high in-plane oriented structure using a directional freeze-drying and mechanical extrusion strategy, and the resulting ionic liquids crystal (ILC)-BNNS/aramid nanofiber/PVA (IBAP) aerogel film has strong anisotropic thermal conductivity. The highly oriented IBAP aerogel film has good thermal stability, flexibility, and mechanical properties. The above study shows that, by designing the orientation arrangement of the thermally conductive fillers and constructing a tightly aligned structure, the mechanical properties and in-plane thermal conductivity of the materials can be improved to achieve the effect of enhancing the properties of the materials.

Polyurethanes (PU) are recognized for their potential as excellent interfacial heat management materials due to their excellent mechanical properties, elasticity, and low-temperature resistance [19,20,21,22]. BN has emerged as a promising inorganic nanomaterial in recent years, known for its excellent thermal conductivity and thermal stability, and is widely used as functional filler for the preparation of high-performance polymer nanocomposites [23,24,25,26]. In this work, we took advantage of the high thermal conductivity of functional BN (FBN), as well as the excellent mechanical properties, elasticity, and low-temperature resistance of PU material [27,28,29,30]. We introduced FBN into PU to prepare an FBN/PU precursor solution and prepared single-layer fiber membrane and multilayer structured composite fiber membrane by layering using the electrostatic spinning technique. A multilayer structured composite fiber membrane was constructed by designing the gradient structure of the fiber membrane to make it have good flexibility, thermal conductivity, and mechanical properties. The practical application of the FBN/PU composite fiber membrane in thermal management was explored, and the membrane has application value in the field for thermal management of wearable electronic devices.

## 2. Results and Discussion

### 2.1. Preparation and Structural Characterization of FBN/PU Composite Fiber Membrane

**Figure 1:**
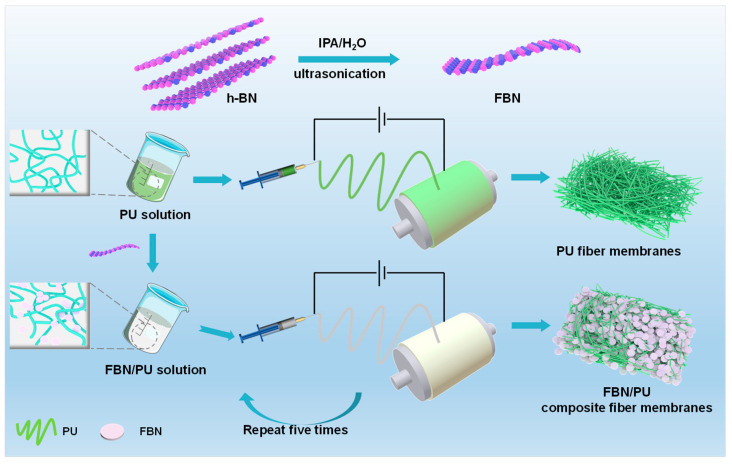
Schematic of the preparation of FBN, PU composite fiber membrane, and FBN/PU composite fiber membrane.

The preparation process of FBN/PU composite fiber membrane and FBN is shown in Figure 1 and described in detail in Section 3. The FBN/PU precursor was spun into a composite fiber membrane with a five-layer structure by electrostatic spinning. The one-dimensional structure of the fibers can be well dispersed in the laminar structure, and the FBN has a homogeneous arrangement in the laminar structure, so the FBN/PU precursor forms a multilayered composite membrane by electrostatic spinning. The FBN/PU composite membrane constructs a laminar network structure, and the composite membranes prepared have promising applications in thermal management. A schematic of the fine structure of FBN was observed with transmission electron microscopy (TEM) (Figure S1). It is possible to visualize the nanosheet layer structure of h-BN after sonication, and h-BN was peeled off into a few layer structures, indicating the successful preparation of FBN after h-BN sonication. After dispersing the ultrasonically complete FBN in water, the Tyndall effect can be seen using a laser pointer (Figure S2), indicating that the FBN can be uniformly dispersed in the aqueous solution. The increase in the dispersion of the FBN also laterally indicates that the ultrasonication experiments have successfully exfoliated the h-BN into fewer layers of FBN, and the Tyndall effect also proves the stability of the FBN.

**Figure 2:**
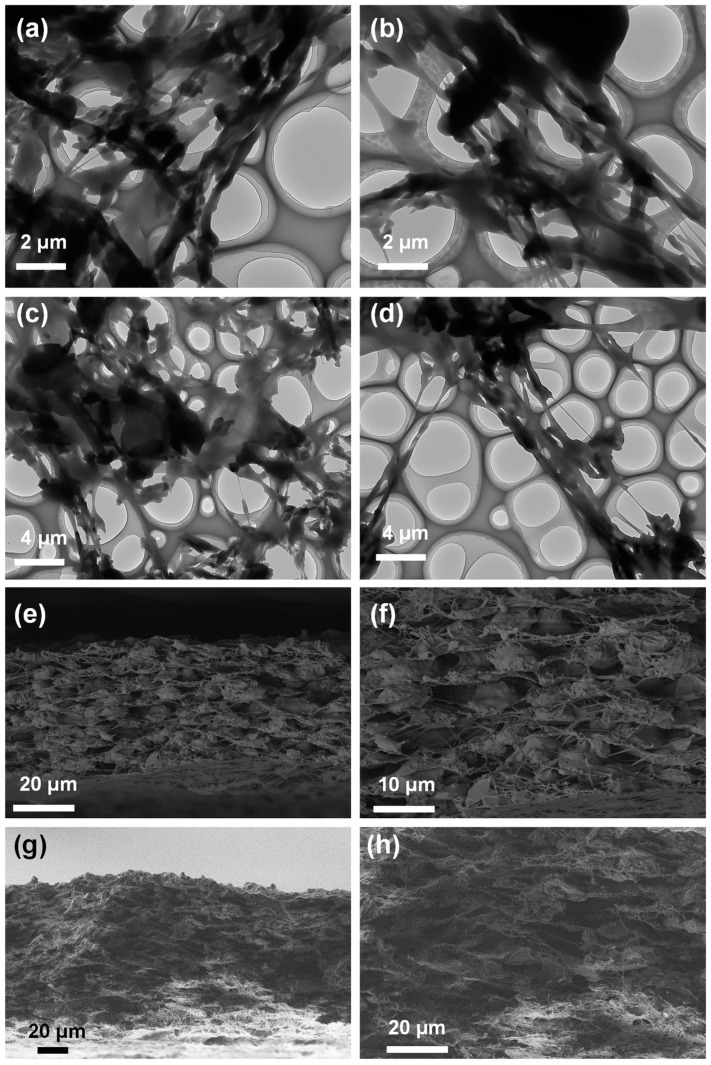
The TEM images (**a**–**d**) of FBN/PU composite fiber membrane. Cross-section SEM images and magnification images of (**e**,**f**) 6-PU and (**g**,**h**) 2-10-FBN/PU composite fiber membrane.

The morphological and structural characteristics of the FBN/PU composite fiber membranes with different gradient structures were analyzed using TEM and SEM (Figure 2 and Figure S3). The TEM images (Figure 2a–d) showed that the sheet-like structure of the FBN/PU composite fiber membranes was piggybacked on ultrafine nanofibers, all connected by single ultrafine fibers. FBN was densely stacked on the PU fiber chain, filling the direct void of the fiber chain, increasing the density of the fiber membrane, which was conducive to the construction of the thermal conductivity pathway. The increase in structural densities improved the mechanical properties of the fiber membrane and allowed it to form a laminar network structure.

Figure S3 shows the surface SEM images of the FBN/PU composite fiber membrane structure. It can be seen that the FBN successfully attached to the PU fibers, the gap between the fibers was significantly reduced, and the surface of the composite fiber membrane became more compact. Figure 2e,f show cross-sectional SEM images of 6-FBN/PU composite fiber membranes and 2-10-FBN/PU composite fiber membranes, respectively, which can show the lamellar structure of multilayer composite fiber membranes. The FBN/PU composite fiber membranes with 2–10 mL dosage gradient arrangement have a very large area of the single-layer membrane, because 2-FBN/PU composite fiber membranes are at the bottom, and the thickness of the single-layer membrane is very similar to that of the fiber membranes with upper layers continuing to be spun. The thickness and area of the single layer of FBN/PU composite fiber membrane are very different from that of the spun fiber membrane, and there is a large gap between the layers of the bottom layer of the fiber membrane, which affects the vertical thermal conductivity of the FBN/PU composite fiber membrane. 2-10-FBN/PU composite fiber membrane arranged in a gradient of the concentration of the single layer of the membrane has a higher area and thickness, and therefore the membrane area is more stable and more tightly connected with the subsequently spun membranes. The gap between each layer of the FBN/PU composite fiber membrane is more stable so that the membrane area is more stable. The gap between each layer of FBN/PU composite fiber membrane is smaller, which is conducive to the improvement of thermal conductivity in the vertical direction.

To further explore the internal structure of monolayer FBN/PU with different thicknesses, IR spectroscopic tests were performed on them. From the IR spectrograms in Figure S4, it can be obtained that all the FBN/PU composite fiber membranes showed characteristic peaks of hydroxyl (-OH) at 3330 cm^−1^, with the characteristic peaks of urethane bonds (-C=O) at 1730 cm^−1^. These characteristic peaks are all the functional groups of PU polymer. Moreover, the characteristic peaks of BN appeared at 1360 cm^−1^ and 800 cm^−1^ in the FBN/PU composite fiber membrane samples, which proved the successful introduction of FBN into the PU composite fiber membrane.

### 2.2. Thermal Conductive Properties and Thermal Conductive Mechanism of FBN/PU Composite Fiber Membrane

**Figure 3:**
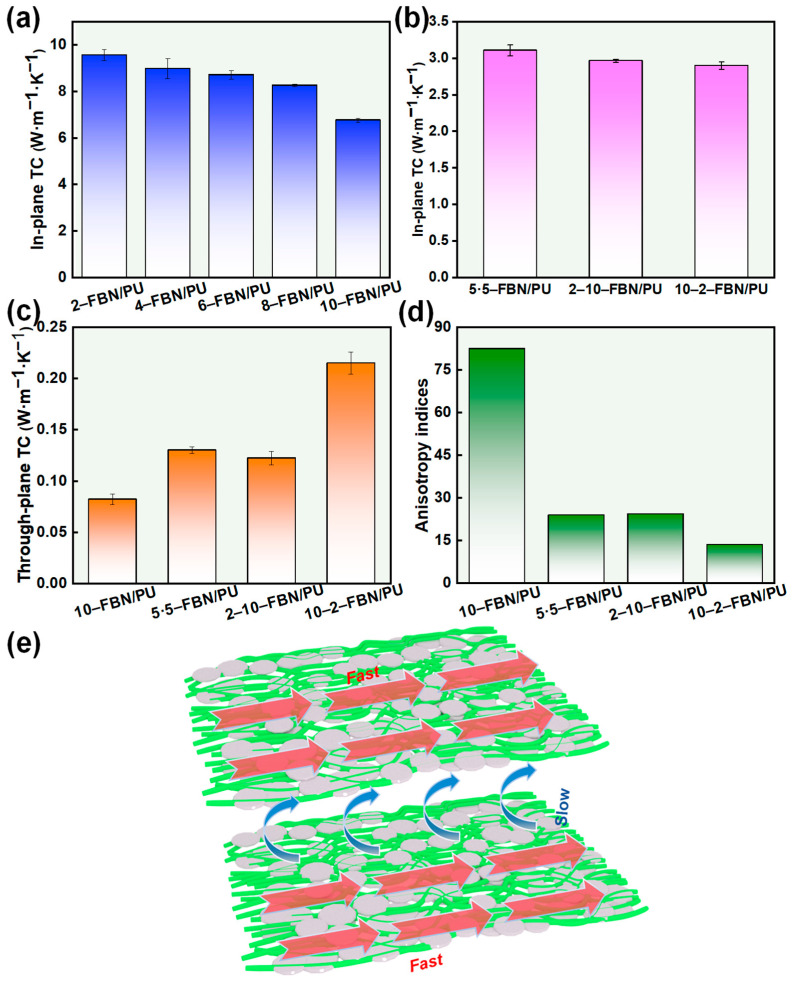
In-plane TC of single-layer FBN/PU composite fiber membrane and PU composite fiber membrane (**a**) and multi-layer FBN/PU composite fiber membrane (**b**). Through-plane TC of FBN/PU composite fiber membrane (**c**). Anisotropy indices (**d**) of FBN/PU composite fiber membrane. (**e**) Thermal conductivity mechanism of FBN/PU composite fiber membranes.

In order to investigate the thermal conductivity of FBN/PU composite fiber membranes with different gradient structure designs, the thermal conductivity of single-layer FBN/PU composite fiber membranes and FBN/PU fiber composite membranes with different gradient structure designs were tested by laser flash (Figure 3), respectively. Due to the amorphous nature of pure PU and the severe scattering of phonons at the chain end of PU and chain entanglement results in poor thermal conductivity of the pure PU fiber membrane. The thermal conductivity of FBN/PU composite fiber membrane was significantly improved by the addition of FBN, which indicated that FBN and the interconnection promoted the formation of heat conduction pathways and reduced the cross-sectional thermal resistance. The thermal conductivity of the spun FBN/PU composite fiber membrane gradually decreases with the increase in the amount of precursor solution of the single-layer FBN/PU composite fiber membrane, because the thickness and volume of the spun FBN/PU composite fiber membrane start to increase with the increase in the amount of FBN/PU precursor solution. With the increase in the volume of the space, the internal orientation of the FBN/PU composite fiber membrane changes with the increase of space volume. The internal orientation of the FBN/PU composite fiber membrane becomes worse, and the heat transfer inside the fiber membrane extends in random directions, with the extension range becoming larger. Therefore, the reduction of the thickness of the single-layer FBN/PU composite fiber membrane increases the in-plane thermal conductivity. In addition, compared with the 5-5-FBN/PU composite fiber membrane, the thickness of the 2-10-FBN/PU composite fiber membrane has been increased without a significant decrease in the in-plane thermal conductivity. This is due to the design of the gradient structure, which makes the FBN more tightly arranged on the fibers, and it is easier to form a continuous thermal conductive pathway. This is because the gradient structure design makes the FBN more tightly arranged on the fibers, which is easier to form a continuous thermal conduction pathway, and the in-plane thermal conductivity of the composite fiber membrane is well preserved [31].

The heat transfer mechanism of FBN/PU film is shown in Figure 3e. FBN and PU fibers form a tightly interconnected laminated structure to construct the thermal conductivity network of FBN filler, which provides a fast conduction channel for heat transfer in the plane direction and promotes heat for conduction along the network in the form of phonons to achieve the high efficiency in heat transfer. However, due to the lateral arrangement of the laminated structure in the plane, the filler has gaps in the vertical direction, which makes it difficult to form a continuous thermal conductive network, thus reducing the thermal conductivity of the material in the vertical direction.

The in-plane thermal conductivity (TC) of previously reported membranes is summarized in Table 1. In comparison with them, the composite fiber membranes we prepared have higher levels of in-plane TCs. This is due to the design of the gradient structure of the composite fiber membrane, where the tightly contacted multilayer structure enhances the in-plane heat conduction paths, which significantly increases the in-plane TC.

### 2.3. Mechanical Properties of FBN/PU Composite Fiber Membrane

**Figure 4:**
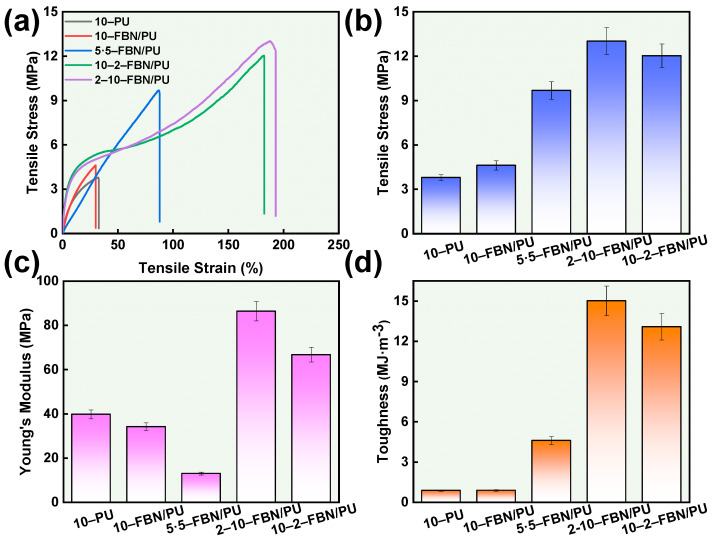
(**a**) Stress–strain curves, (**b**) tensile stress, (**c**) Young’s modulus, and (**d**) toughness of FBN/PU composite fiber membrane.

The FBN/PU composite fiber membranes have excellent mechanical properties and flexibility, and the prepared FBN/PU composite fiber membranes can be cut into long strips and blocks. They can be folded, curled, and restored to their original shape, which indicates that the fiber membranes have good flexibility (Figure S5). The stress–strain curves and mechanical properties of FBN/PU composite fiber membranes with different gradient structure designs are shown in Figure 4, which shows that the gradient structure design of 10-2-FBN/PU composite fiber membranes and 2-10-FBN/PU composite fiber membranes has increased thickness, leading to greater tensile strength and higher elongation at the break. In addition, the stress, Young’s modulus, and toughness of FBN/PU composite fiber membrane with gradient structure design are higher than those of FBN/PU composite fiber membrane with uniform arrangement and monolayer, which indicates that the design of gradient structure greatly enhances the mechanical properties of the FBN/PU composite fiber membrane. The comprehensive performance of the 2-10-FBN/PU composite fiber membrane reaches the highest strength, Young’s modulus, and toughness up to 12.03 MPa, 86.37 MPa, and 15.02 MJ·m^−3^, respectively. The FBN/PU composite fiber membrane can be suitable for various environments and has the potential for the application of wearable electronic devices. In particular, the 2-10-FBN/PU composite fiber membranes greatly improve the mechanical properties of the membranes while retaining the thermal conductivity of the monolayer FBN/PU composite fiber membranes. This enables the 2-10-FBN/PU composite fiber membranes to achieve a certain degree of simultaneous improvement in thermal conductivity and mechanical properties. This proves the superiority of the gradient structure design.

### 2.4. Demonstration of FBN/PU Composite Fiber Membrane in Practical Application

**Figure 5:**
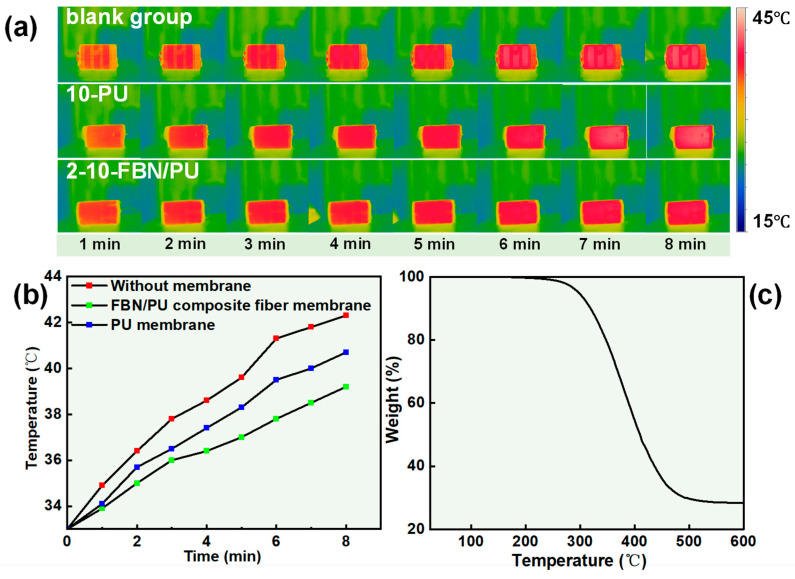
(**a**) Temperature variations of the battery during operation. (**b**) Time–temperature curve of battery operation. (**c**) TG curves for 2-10-PU/FBN composite fiber membrane.

To further determine the practical use of the composite membranes and to better simulate the practical application of FBN/PU composite fiber membranes in wearable electronic devices, heat dissipation tests were conducted using different FBN/PU composite fiber membranes (Figure S6). A pure PU membrane and a 2-10-FBN/PU composite fiber membrane were used as the heat dissipation membranes for the battery, and a blank group was added for the control test. With the same dimensions of the fiber membrane, the hot spot temperature of the battery was recorded by using an infrared (IR) thermal imaging camera. The temperature recording was carried out once every minute until the temperature stabilized, the IR thermal imaging images were taken, and the warming curves were plotted. The IR thermal images and the corresponding time–temperature curves are shown in Figure 5a,b, respectively. The 2-10-FBN/PU composite fiber membrane has the lowest equilibrium temperature, the slowest rate of warming, and the best heat dissipation ability, with a temperature reduction of 3 °C compared to the blank experimental group, and a reduction of 1.5 °C compared to the pure PU membrane, which demonstrates the superiority of its heat dissipation ability. The application of actual heat dissipation proves the excellent heat dissipation performance of the 2-10-FBN/PU composite fiber membrane, which has the prospect of application in the field of heat dissipation of electronic devices.

The thermal stability of composite films in high-temperature environments is critical for their practical applications. To test the thermal stability of the FBN/PU composite fiber membranes, TGA tests were performed on the 2-10-FBN/PU composite fiber membranes. As shown in Figure 5c, the 2-10-FBN/PU composite fiber membrane started to undergo thermal decomposition at 300 °C, which was attributed to the decomposition of the PU polymer. When the temperature exceeded 460 °C, the weight remained stable with increasing temperature, and the residual mass was 28%, which was attributed to the incorporation of FBN and resulted in a residual mass at equilibrium proportional to the amount of FBN. Thus, the thermal study clearly shows that the incorporation of FBN significantly increases the thermal stability of the FBN/PU composite fiber membrane. The prepared FBN/PU composite fiber membrane has good thermal stability and can be adapted to thermal management work in higher-temperature environments.

## 3. Materials and Methods

### 3.1. Materials

TPU (thermoplastic polyurethane) was acquired from Qingdao Pansi Technology Co., Ltd. (Qingdao, China). h-BN was acquired from ESK Ceramics GmbH & Co., (Kempten, Germany). DMF (99.5%. N, N-Dimethylformamide) was acquired from Sinopharm Chemical Reagent CO., Ltd. (Shanghai, China) Isopropanol (IPA, 98.5%) was obtained from Sinopharm Chemical Reagent CO., Ltd.

### 3.2. Methods

#### 3.2.1. Preparation of FBN

A total of 2.4 g of h-BN and 100 mL of IPA in 100 mL of deionized water were mixed in a beaker to form a solution and stirred at 500 rpm for 35 min, and upon completion of the stirring, the mixture was subjected to sonication for 6 h. After the completion of sonication, the solution was subjected to two centrifugations, with the first at 500 rpm for 5 min—the supernatant was removed, and the supernatant was subjected to a second centrifugation—and the second at 10,000 rpm for 10 min. At the end of centrifugation, the lower precipitate was taken and dried to obtain FBN.

#### 3.2.2. Preparation of FBN/PU Precursor Solution

In a small beaker, 2 g of PU and 30 mL of DMF were added; the small beaker was placed into a magnetic water bath, magnets were added, and this was stirred at 70 °C. After the PU was completely dissolved, 0.5 g of FBN powder was added to the PU solution and heated with stirring at 70 °C for 0.5 h. The FBN/PU solution was then placed in an ultrasonic cleaner for 2 h to more completely disperse the FBN in the PU solution. Subsequently, the FBN/PU solution was put into a vacuum oven for vacuum defoaming to obtain the FBN/PU precursor solution.

#### 3.2.3. Preparation of FBN/PU Composite Fiber Membrane

The FBN/PU precursor solution was extracted with a syringe, and then the FBN/PU precursor solution was electrostatically spun in accordance with the parameters in Table S1, and the five-layer composite fiber membranes were prepared layer by layer. After the completion of the fiber membrane spinning, the oven temperature was adjusted to 35 °C with the composite fiber membrane for 4 h, so that the solvent evaporated completely, and then the fiber membrane was demolded and cut. Five single-layer composite fiber membranes, a 10 mL–2 mL cis-content gradient composite fiber membrane, a 2 mL–10 mL counter-content gradient composite fiber membrane, and a 5 mL uniformly aligned composite fiber membrane were prepared with this method, respectively. Subsequently, various performance tests were conducted. The 2–10 mL monolayer composite fiber membranes were named (2, 4, 6, 8, 10)-FBN/PU membranes, with thicknesses of 0.014 mm, 0.031 mm, 0.048 mm, 0.051 mm, 0.056 mm, respectively. In addition, 10 mL–2 mL cis-content-gradient composite fiber membranes, 2 mL–10 mL inverse-content-gradient composite fiber membranes, and 5 mL of homogeneously aligned composite fiber membranes were named (2-10, 5-5, 10-2)-FBN/PU composite fiber membranes, with thicknesses of 0.132 mm, 0.135 mm, 0.104 mm, respectively.

### 3.3. Characterization

The fabricated FBN/PU composite fiber membranes were tested using a Fourier infrared spectrometer from 500 to 4000 cm^−1^ (FTIR, Niciletis20, Thermo Fisher Scientific, Waltham, MA, USA). The morphologies of the FBN and FBN/PU composite fiber membranes were observed using transmission electron microscope (TEM, JEM-1400Flash, JEOL Ltd., Tokyo, Japan). The surface and cross-section morphology images of the FBN/PU composite fiber membrane were obtained using scanning electron microscope(SEM, Sigma500, Sigma-Aldrich, Darmstadt, Germany). The heat diffusion coefficients of FBN/PU composite fiber membranes of different gradient structural designs were tested using an LFA thermal conductivity meter (LFA 467 HyperFlash, NETZSCH, Selb, Germany). The thermal stability from 25 to 600 °C (10 °C/min, nitrogen) of the FBN/PU composite membranes produced by electrostatic spinning and the weight change before and after thermal decomposition were tested using thermogravimetric analysis (TG209, NETZSCH, Germany). Strain was applied to FBN/PU composite fiber membranes, and dynamic mechanical analysis was performed using a universal material testing machine (E1000, Instron Corporation, Norwood, MA, USA).

## 4. Conclusions

In summary, FBN/PU composite fiber membranes were prepared by combining FBN with high thermal conductivity and PU with excellent mechanical properties using electrostatic spinning. The structure design of the FBN/PU composite fiber membranes was carried out, and the effect of the gradient structure design of the fiber membranes on the thermal conductivity and mechanical properties of the FBN/PU composite fiber membranes was investigated. Benefitting from the gradient structure design of the 2–10-FBN/PU composite fiber membrane, the in-plane thermal conductivity, mechanical properties and thermal stability were balanced, and the thermal conductivity (2.96 W·m^−1^·K^−1^) and mechanical properties were analyzed (tensile strength, Young’s modulus, and toughness reaching up to 12.03 MPa, 86.37 MPa and 15.02 MJ·m^−3^, respectively). The thermal conductivity in the vertical direction reached 0.122 W·m^−1^·K^−1^. In addition, the thermal management of the FBN/PU on the practical application of wearable electronic devices was confirmed. The design of thermally managed, high-mechanical performance FBN/PU composite fiber membranes provides new insights for expanding the wide range of applications of composite membranes in wearable electronics.

## Figures and Tables

**Table 1:** Comparison of in-plane TC of our FBN/PU composite fiber membrane with other thermally conductive polymeric materials.

Matrix	Filler	Filler Content	In-Plane TC (W·m^−1^·K^−1^)	Reference
TPU	BNNSs	30 wt%	1.80	[32]
PU	BNNS/CNTs	20 wt%	1.49	[33]
TPU	RGO	1.04 wt%	0.80	[34]
Epoxy	3D-BN	28.1 wt%	1.85	[35]
PU	BN	70 wt%	0.653	[36]
PU	BN-Ag	2.5 wt%	0.84	[28]
TPU	Lys@BNNSs	3 wt%	0.52	[28]
Epoxy	BN	17.57 wt%	0.65	[37]
PEEK	n-BN/m-BN	40 wt%	1.77	[38]
TPU	BNNSs	25 wt%	0.844	[39]
PU	FBN	20 wt%	2.96	This Work

## Data Availability

The data presented in this study are available upon request from the corresponding author.

## Supplementary Materials

The following supporting information can be downloaded at: https://www.mdpi.com/article/10.3390/molecules30071449/s1, Figure S1. The TEM images of FBN; Figure S2. The Tyndall Effect of FBN; Figure S3. Surface SEM images of (a) 10-PU, (b) 2-FBN/PU, (c) 4-FBN/PU, (d) 10-FBN/PU, (e) 2-10-FBN/PU, (f) 10-2-FBN/PU composite fiber membrane; Figure S4. FTIR of Single layer FBN/PU composite fiber membrane; Figure S5. FBN/PU film can be rolled and folded; LFA Measurement. Figure S6. Schematic diagrams of Li-ion batteries without external coating and with FBN/PU membrane. Table S1. Parameters of electrostatic spinning machine.
