# Supplementary material for: Gradient Structure Construction of High Thermal Conductivity Polyurethane/Boron Nitride Composite Fiber Membrane for Thermal Management
Source: Molecules. 2025 Mar 25;30(7):1449. doi: 10.3390/molecules30071449 (PMC11990400; doi:10.3390/molecules30071449)
Supplement: Supplementary file 1 [file molecules-30-01449-s001.zip › molecules-3527909-supplementary.pdf]

Supplementary Information

Gradient Structure Construction of High Thermal Conductivity Polyurethane/Boron Nitride Composite Fiber Membrane for Thermal Management

Zhengyang Miao [1,2], Jingwei Li [1], Yidan Liu [1] and Fang Jiang [1,2,3,*]

1 State Key Laboratory of Bio-Based Fiber Materials, Zhejiang Sci-Tech University, Hangzhou 310018, China; mzy1147911302@163.com (Z.M.); ljw1263360689@126.com (J.L.); liuyidan@zstu.edu.cn (Y.L.)

2 Shaoxing-Keqiao Institute, Zhejiang Sci-Tech University, Shaoxing 312000, China

3 Faculty of Science, Shanghai University, 99 Shangda Road, Shanghai 200444, China

* Correspondence: jiangfang@zstu.edu.cn

Table S1. Parameters of electrostatic spinning machine.

Setting	Parameters
Voltage	20.00 KV
Roller Speed	500 r/min
Pusher speed	0.03 ml/s

LFA Measurement

The thermal diffusivity of monolayer FBN/PU composite fiber membranes and FBN/PU composite fiber membranes designed with gradient structure was analyzed by laser flash. The samples were cut into 12.7 mm diameter discs for testing.

The in-plane thermal conductivity was calculated by

$K = \alpha \times \rho \times Cp$

where α is the in-plane thermal diffusivity, ρ is the density and Cp is the specific heat capacity.

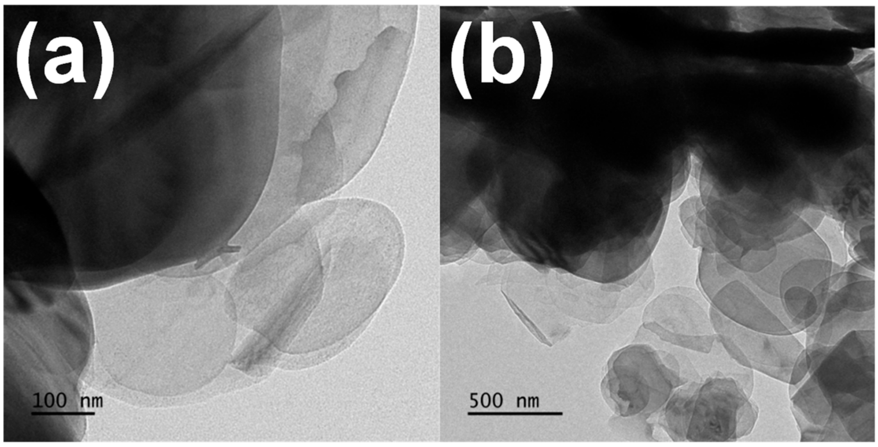

Figure S1. The TEM images of FBN

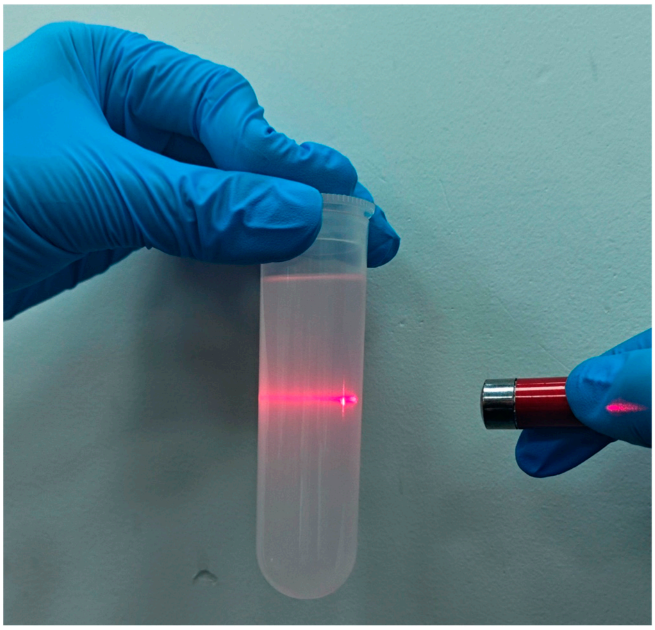

Figure S2. The Tyndall Effect of FBN.

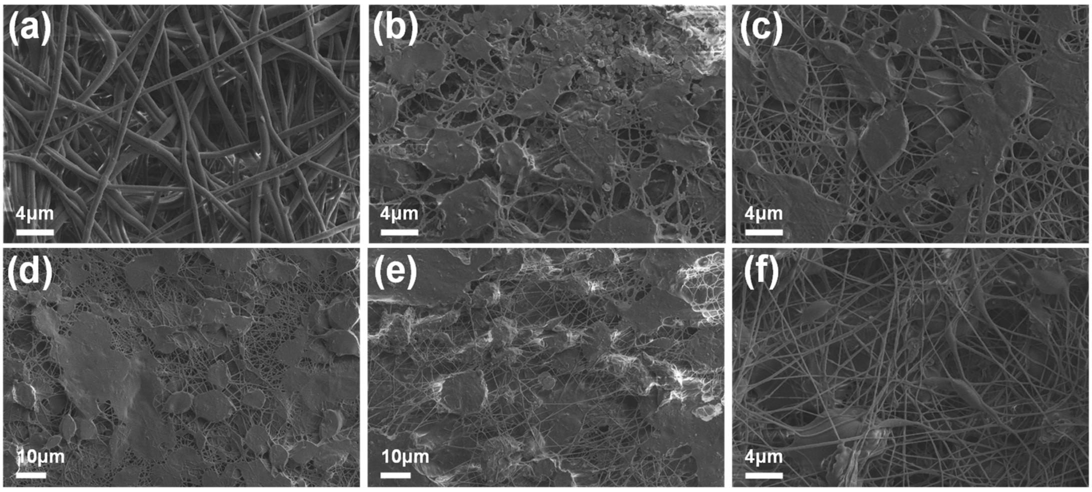

Figure S3. Surface SEM images of (a) 10-PU; (b) 2-FBN/PU; (c) 4-FBN/PU; (d) 10-FBN/PU; (e) 2-10-FBN/PU; (f) 10-2-FBN/PU composite fiber membrane.

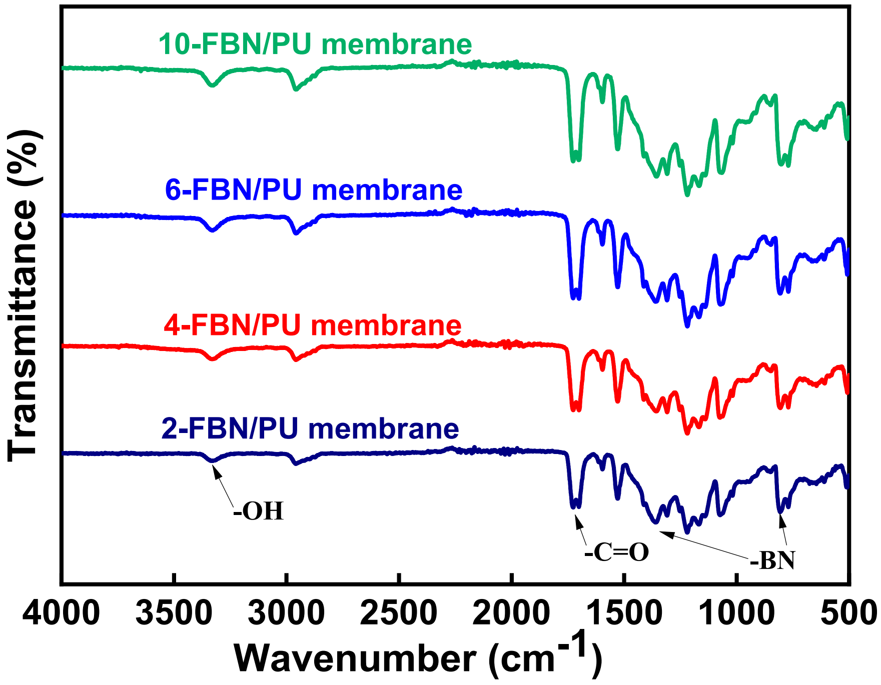

Figure S4. FTIR of Single layer FBN/PU composite fiber membrane.

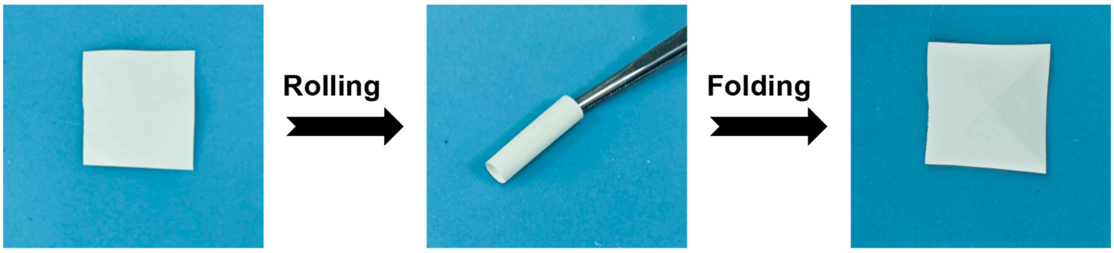

Figure S5. FBN/PU film can be rolled and folded.

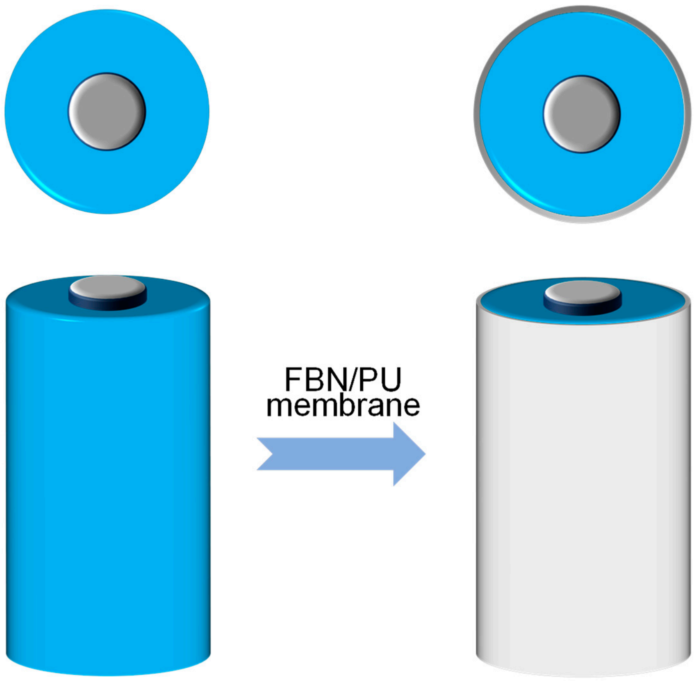

Figure S6. Schematic diagrams of Li-ion batteries without external coating and with FBN/PU membrane.
